# Finerenone’s Impact on Major Adverse Cardiovascular Events in Chronic Kidney Disease and Type 2 Diabetes Mellitus: A Systematic Review

**DOI:** 10.7759/cureus.68274

**Published:** 2024-08-31

**Authors:** Vignesh Murugan, Farhana Nazmin, Jian Garcia, Sanjana Singareddy, Surakchhya Dhakal, Therese Anne Limbaña, Safeera Khan

**Affiliations:** 1 Department of Research, California Institute of Behavioral Neurosciences and Psychology, Fairfield, USA

**Keywords:** drug-induced hyperkalemia, estimated glomerular filtration rate (egfr), chronic kidney disease (ckd), diabetes mellitus type 2, mineralocorticoid receptor antagonist, major adverse cardiovascular events, finerenone

## Abstract

Chronic kidney disease (CKD) impacts about 10% of adults globally and substantially elevates the risk of major adverse cardiovascular events (MACE), such as heart attacks, strokes, cardiovascular-related deaths, and hospital admissions due to heart failure. The interplay between CKD and cardiovascular disease (CVD) leads to poor health outcomes. Nevertheless, there is a scarcity of systematic reviews focusing on the effectiveness of finerenone, a new non-steroidal mineralocorticoid receptor antagonist (MRA), in lowering these risks. In this systematic review, we aim to evaluate the impact of finerenone on reducing MACE in individuals with CKD and type 2 diabetes mellitus (T2DM). CKD pathophysiology involves hyperglycemia, hypertension, and dyslipidemia, leading to glomerular hyperfiltration, inflammation, and fibrosis. Traditional treatments, including angiotensin-converting enzyme inhibitors (ACEi), angiotensin II receptor blockers (ARBs), and sodium-glucose cotransporter-2 inhibitors (SGLT2i), often fall short in preventing cardiovascular events. Steroidal MRAs like spironolactone and eplerenone, while effective in reducing proteinuria, are limited by hyperkalemia risks. Finerenone offers a more selective mechanism, reducing sodium retention, inflammation, and fibrosis, with a lower risk of hyperkalemia. We searched five electronic databases comprehensively, identifying studies consistently demonstrating that finerenone significantly reduces MACE and improves renal outcomes by reducing albuminuria and slowing the fall in estimated glomerular filtration rate (eGFR). However, limitations include study heterogeneity, short follow-up periods, and potential publication bias. In conclusion, finerenone shows promise as a therapeutic option for CKD and T2DM, reducing MACE and improving renal outcomes. Further research is needed to understand its long-term benefits and safety across diverse populations.

## Introduction and background

“Every life lost to a preventable cardiovascular event is a tragedy, a challenge, and an urgent call for better care.” This statement highlights the urgent necessity of addressing cardiovascular events, especially in high-risk groups like individuals with chronic kidney disease (CKD). Compared to the healthy population, patients with CKD face a markedly higher rate of major adverse cardiovascular events (MACE), such as heart attacks, strokes, cardiovascular deaths, and hospitalizations for heart failure. This disparity stresses the urgent need for effective interventions.

CKD is a cumulative condition characterized by a steady decline in kidney function over a period of time. It is categorized into stages based on the glomerular filtration rate (GFR), with stages 1-4 indicating mild to severe kidney impairment and stage 5 representing kidney failure. CKD impacts roughly 10% of adults globally [1]​​. This condition not only compromises kidney function but also dramatically elevates the risk of cardiovascular disease (CVD). The interplay between CKD and CVD creates a cycle where one condition exacerbates the other, leading to poor health outcomes. MACE are particularly severe outcome for CKD patients, often resulting in decreased quality of life and increased healthcare costs​​​​ [2].

Recent advancements in the management of CKD and its cardiovascular complications have introduced new therapeutic options. Traditional treatments have focused on controlling hypertension, managing diabetes, and reducing cholesterol levels. However, these measures alone are often insufficient to prevent cardiovascular events in CKD patients. The introduction of mineralocorticoid receptor antagonists (MRAs) represents a significant advancement in this field. MRAs like spironolactone and eplerenone reduce proteinuria and slow kidney disease progression. However, their risk of causing hyperkalemia limits their use​​ [3].

Finerenone, a novel non-steroidal MRA, has emerged as a promising therapeutic option for CKD patients with cardiovascular comorbidities. Unlike traditional MRAs, finerenone has a more selective mechanism of action, which reduces the risk of hyperkalemia while providing significant cardiorenal benefits​​​​ [4]. Randomized controlled trials (RCTs), such as the FIDELIO-DKD and FIGARO-DKD trials, have demonstrated that finerenone can effectively reduce the incidence of adverse cardiac events in type 2 diabetes mellitus (T2DM) and CKD patients. These studies have shown that finerenone lowers the risk of MACE and slows the progression of kidney disease, making it a dual-benefit therapy for this high-risk population​​​​ [5].

Despite these advancements, significant knowledge gaps remain regarding finerenone’s full potential and limitations in CKD patients with cardiovascular comorbidities. While existing studies provide good evidence of the efficacy of finerenone in reducing the progression of CKD, data on its ability to reduce MACE still need to be provided. Additionally, the impact of finerenone on specific cardiovascular outcomes, such as heart failure and arrhythmias, requires further investigation.

Our systematic review aims to address these gaps by comprehensively analyzing the existing literature on the efficacy of finerenone in reducing adverse cardiovascular outcomes in patients with CKD. By synthesizing data from multiple high-quality RCTs, we aim to understand better how finerenone can be integrated into standard care to reduce the incidence of MACE in this vulnerable population. This systematic review will contribute valuable insights to the current body of knowledge and provide evidence-based recommendations for clinicians managing CKD patients at high risk of cardiovascular events. This review will explore the therapeutic efficacy of finerenone and identify areas where further research is needed, ultimately guiding future clinical practice and improving patient outcomes.

## Review

Methods

This systematic review followed the Preferred Reporting Items for Systematic Reviews and Meta-Analyses (PRISMA) guidelines. The objective was to assess the efficacy of finerenone in decreasing the occurrence of MACE in patients with CKD and CVD in comparison to standard care alone.

Search Strategy

A thorough search strategy was devised and executed across five electronic databases: PubMed, PubMed Central (PMC), Cochrane Library, Science Direct, and MDPI. The search terms comprised multiple combinations of keywords and medical subject headings (MeSH) related to CKD, CVD, and finerenone (Table 1).

**Table 1:** Search strategy with keywords

Search strategy	Database/search strategy used	Number of papers identified
(“chronic kidney disease” OR “CKD” OR “kidney failure” OR “renal insufficiency”) AND (“cardiovascular disease” OR “CVD” OR “heart disease” OR “coronary artery disease” OR “heart failure” OR “stroke”) AND (“finerenone”)	PubMed Routine	147
((“Naphthyridines/therapeutic use”[Majr]) AND (“Kidney Failure, Chronic/complications”[Majr] OR “Kidney Failure, Chronic/drug therapy”[Majr]))	PubMed MeSH	3
((“finerenone”[Title/Abstract] OR “BAY 94-8862”[Title/Abstract]) AND (“chronic kidney disease” OR “CKD” OR “kidney failure” OR “renal insufficiency”)) AND (“cardiovascular diseases” OR “CVD” OR “heart disease” OR “coronary artery disease” OR “heart failure” OR “stroke”)	PubMed Advanced	144
(((“finerenone”[Title] OR “BAY 94-8862”[Title])) AND (“chronic kidney disease” OR “CKD” OR “kidney failure” OR “renal insufficiency”)) AND (“cardiovascular diseases” OR “CVD” OR “heart disease” OR “coronary artery disease” OR “heart failure” OR “stroke”)	PMC	98
“chronic kidney disease” OR “CKD” OR “kidney failure” OR “renal insufficiency” in Title Abstract Keyword AND “cardiovascular disease” OR “CVD” OR “heart disease” OR “coronary artery disease” OR “heart failure” OR “stroke” in Title Abstract Keyword AND “finerenone” OR “BAY 94-8862” in Title Abstract Keyword	Cochrane Library	108
Title, abstract, keywords: (“finerenone” OR “BAY 94-8862”) Text word: (“chronic kidney disease” OR “CKD” OR “renal insufficiency”) AND (“cardiovascular disease” OR “CVD” OR “heart disease” OR “coronary artery disease” OR “heart failure” OR “stroke”)	Science Direct	87
Title / Keyword: “finerenone”	MDPI	23

Screening of Articles

We initially collected 610 articles, eliminated duplicates, and reviewed the remaining papers based on their titles, abstracts, and full-text content. Subsequently, we applied quality assessment tools to nine selected research papers.

Inclusion Criteria

For inclusion, studies had to involve patients with CKD stages 1-4 and concurrent CVD, where the intervention was finerenone added to standard care. The comparison group included patients receiving either standard care alone or a placebo. Eligible studies were required to report on the incidence of MACE, which includes myocardial infarction, stroke, cardiovascular death, and hospitalization for heart failure. We included only RCTs, systematic reviews, and meta-analyses published in English.

Exclusion Criteria

We defined exclusion criteria to ensure the relevance and quality of the included studies. We excluded studies involving patients with end-stage renal disease (ESRD) on dialysis, those with isolated CKD or CVD, and studies where finerenone was not the primary intervention or was combined with other investigational drugs. Additionally, we excluded studies without a clear comparison group, those not reporting MACE, and those focusing solely on secondary outcomes. We also excluded non-randomized trials, reviews, case reports, and articles not published in English.

Quality Assessment

We imported all identified records into Microsoft Excel and removed duplicates. We then conducted a two-stage screening process on the remaining records. First, we screened articles based on their titles and excluded irrelevant studies. Next, we reviewed the abstracts of the remaining articles to determine their eligibility, excluding those that did not meet the inclusion criteria. We retrieved and evaluated full-text articles from the remaining studies for eligibility according to the inclusion and exclusion criteria. Data were extracted using a standardized form to capture information on study design, population characteristics, interventions, comparisons, outcomes, and key results. We then used the Cochrane Risk of Bias 2 (RoB 2) tool to assess the quality of the RCTs (Table 2). The AMSTAR 2 (A Measurement Tool to Assess Systematic Reviews) checklist was used for the two meta-analyses (Table 3). Most studies demonstrated a low risk of bias across critical domains, although some showed unclear risk in areas such as blinding and selective reporting.

**Table 2:** A quality appraisal using the Cochrane bias assessment tool + indicates yes, - indicates no, and ? indicates not clear

Author	Type of Study	Random sequence generation (selection bias)	Allocation Concealment (selection bias)	Blinding of participants and personnel	Blinding of outcome assessment	Incomplete outcome data	Selection report	Other bias
Koya et al., 2023 [6]	Randomized controlled trial	+	+	+	+	+	+	+
Agarwal et al., 2021 [7]	Randomized controlled trial	?	?	+	+	+	?	+
Pitt et al., 2021 [8]	Randomized controlled trial	?	+	+	+	+	+	+
Filippatos et al., 2021 [9]	Randomized controlled trial	+	+	+	+	+	+	+
Bansal et al., 2024 [10]	Randomized controlled trial	?	?	+	+	+	?	+
Filippatos et al., 2022 [11]	Randomized controlled trial	?	?	+	+	+	+	+
Filippatos et al., 2022 [12]	Randomized controlled trial	?	+	+	+	+	+	+

**Table 3:** A quality appraisal using the AMSTAR-2 checklist PICO is an acronym for Patient, Intervention, Comparison, and Outcome; PRISMA: Preferred Reporting Items for Systematic Reviews and Meta-Analyses; AMSTAR: A Measurement Tool to Assess Systematic Reviews

Study characteristic	Jyotsna et al., 2023 [13]	Abdelazeem et al., 2022 [14]
Did the research questions and inclusion criteria incorporate the components of PICO?	Yes	Yes
Did the review report explicitly state that the methods were established before the review, and were any significant deviations from the protocol justified?	The review mentions that it follows the guidelines set forth by the PRISMA statement. However, there is no explicit statement that the review methods were outlined before the review was conducted. Additionally, the report does not justify any deviations from the protocol​.	The review mentions that it follows the guidelines set forth by the PRISMA statement. However, there is no explicit statement that the review methods were outlined before the review was conducted. Additionally, the report does not justify any deviations from the protocol​.
Did the review authors explain their selection of the study designs for inclusion in the review?	Yes	Yes
Did the review authors employ a detailed literature search strategy?	Partially yes - They searched at least two databases, provided keyword and/or search strategy, and justified publication restrictions. However, they have not consulted content experts in the field.	Partially yes - They searched at least two databases, provided keyword and/or search strategy, and justified publication restrictions. However, they have not consulted content experts in the field.
Did the review authors conduct the study selection in duplicate?	Yes	No
Did the review authors conduct the data extraction in duplicate?	Yes	No
Did the authors provide a list of studies that they excluded studies with justifications?	No	No
Were the included studies described in good detail?	Yes	Yes
Did the authors use a good method for evaluating the risk of bias (RoB) in the studies included in the review?	Yes	No
Did the review authors report on the funding sources for the studies included?	No	No
If a meta-analysis was conducted, did the authors use appropriate methods to combine the statistical results?	Yes	Yes
If a meta-analysis was conducted, did the review authors evaluate the potential impact of the risk of bias (RoB) in the individual studies on the outcome of the meta-analysis or other evidence synthesis?	Yes	No
Did the review authors consider the risk of bias in individual studies when interpreting and discussing the results of the review?	Yes	Yes
Did the review authors offer a satisfactory explanation for any heterogeneity observed in the review results?	Yes	Yes
If a quantitative synthesis was performed, did the review authors adequately investigate publication bias (small study bias) and discuss its potential impact on the review results?	Yes	No
Did the review authors disclose any sources of conflict of interest or any funding they received for conducting the review?	Yes	Yes

The goal of this systematic review is to provide a detailed analysis of the current evidence on the efficacy and safety of finerenone in reducing MACE in patients with CKD and CVD. This review seeks to contribute valuable insights and evidence-based recommendations for managing this high-risk patient population by addressing existing knowledge gaps and informing clinical practice.

Results 

Study Selection 

**Figure 1:**
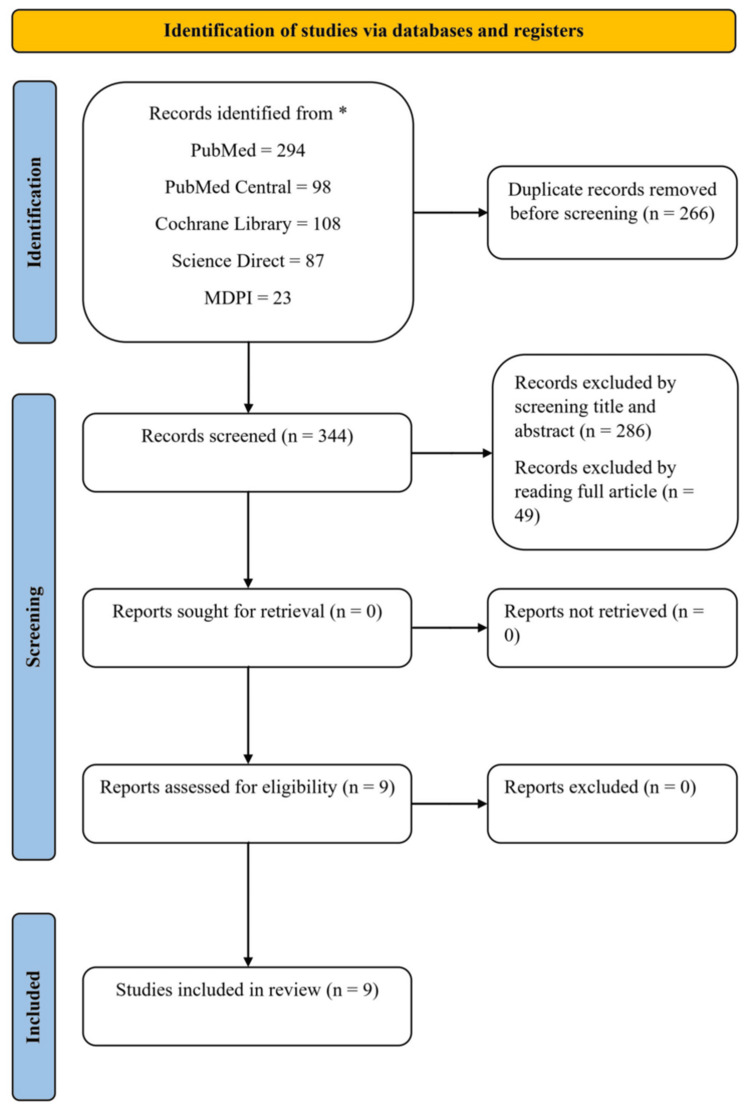
PRISMA 2020 flowchart illustrating the method for article selection *Studies selected based on predefined inclusion and exclusion criteria established before screening PRISMA: Preferred Reporting Items for Systematic Review and Meta-Analysis

We identified a total of 610 articles through the comprehensive database search. After removing 266 duplicates, we screened 344 articles by title and abstract. We excluded 286 articles based on relevance, leaving 58 articles for full-text review. After reviewing the full texts, we excluded 49 articles because they did not directly compare finerenone to standard care, did not focus on major cardiovascular events, or were not RCTs. This process led to the selection of nine articles for the final systematic review (Figure 1).

Study Characteristics

The nine studies included in this review consisted of seven RCTs and two meta-analyses. These studies varied in population characteristics, study duration, interventions, and endpoints. Table 4 provides a rundown of the key characteristics of the included studies.

**Table 4:** Summary of RCT and meta-analyses RCT: randomized controlled trial; MACE: major adverse cardiovascular events; CKD: chronic kidney disease; T2DM: type 2 diabetes mellitus; HF: heart failure; ASCVD: atherosclerotic cardiovascular disease; FIDELIO-DKD: Finerenone in Reducing Kidney Failure and Disease Progression in Diabetic Kidney Disease. FIDELITY: an analysis of two trials - Finerenone in Reducing Kidney Failure and Disease Progression in Diabetic Kidney Disease (FIDELIO-DKD) and Finerenone in Reducing Cardiovascular Mortality and Morbidity in Diabetic Kidney Disease (FIGARO-DKD)

Author and Year of Publication	Drug Studied/Intervention Studied	Number of Patients	Type of Study	Result	Conclusion
Koya et al., 2023 [6]	Finerenone	Post hoc analysis of FIDELIO-DKD - 1,327	RCT	Improved cardiorenal outcomes in Asian patients	Finerenone produces similar cardiorenal benefits in Asian and non-Asian patients
Agarwal et al., 2021 [7]	Finerenone	13,026 (FIDELITY pooled analysis)	RCT	Reduced cardiovascular and kidney outcomes	Finerenone is beneficial for both cardiovascular and kidney outcomes
Pitt et al., 2021 [8]	Finerenone	7,437	RCT	Reduced cardiovascular events	In patients with T2DM and either stage 2 to 4 CKD with moderately elevated albuminuria or stage 1 or 2 CKD with severely elevated albuminuria, finerenone therapy improved cardiovascular outcomes
Filippatos et al., 2021 [9]	Finerenone	5,674	RCT	Reduced cardiovascular outcomes	Finerenone lowered the incidence of composite cardiovascular outcomes, with no evidence of variation in treatment effect based on preexisting cardiovascular disease status
Bansal et al., 2024 [10]	Finerenone	Post hoc analysis - 13,026	RCT	Age and sex-specific outcomes	Finerenone’s benefits are consistent across age and sex
Filippatos et al., 2022 [11]	Finerenone	Subgroup analysis of FIDELIO-DKD - 5,674	RCT	Benefits in patients with and without HF	Finerenone improved cardiorenal outcomes in patients with CKD and T2DM, irrespective of baseline HF history
Filippatos et al., 2022 [12]	Finerenone	13,026 (FIDELITY pooled analysis)	RCT	Reduced cardiovascular and kidney outcomes	Finerenone consistently reduced the risk of cardiovascular and kidney outcomes across the spectrum of CKD in patients with T2DM, regardless of the presence of ASCVD
Jyotsna et al., 2023 [13]	Finerenone	39,995	Meta-analysis	Significant reduction in MACE	Finerenone is effective in reducing cardiovascular events in CKD and T2DM
Abdelazeem et al., 2022 [14]	Finerenone	13,847	Meta-analysis	Significant reduction in cardiovascular events	Among patients with CKD and T2DM, finerenone is associated with lower risks of CV events and heart failure hospitalizations compared with a placebo

Participant Characteristics and Study Outcomes

The nine studies included in this review collectively analyzed data from overlapping cohorts, primarily derived from the FIDELIO-DKD and FIGARO-DKD trials [5,8]. To avoid double-counting participants, we focus on the unique patient groups studied. Jyotsna et al. and Abdelazeem et al. are meta-analyses, so we do not include those in the total sample size calculation [13,14]. The unique cohort from the largest included study, the FIDELITY analysis, involved 13,026 patients, so that will be our total pooled sample size [12]. The mean age of participants was approximately 65 years, and around 70.3% were male. The studies focused on patients with CKD stages 1-4 and concurrent CVD, specifically targeting the reduction of MACE.

Impact on MACE

The studies varied in population sizes, with the smallest being Koya et al. with 1,327 patients and the largest being Agarwal et al. with 13,171 patients [6,7]. The studies involved patients with CKD and T2DM, focusing on those at high risk of MACE. All studies had a median follow-up duration of 2.6 to 2.9 years, providing a relatively consistent period for assessing long-term outcomes. All studies primarily aimed to reduce the incidence of MACE, with some studies also focusing on secondary outcomes, including kidney function and hospitalization rates for heart failure [7-9]. Finerenone significantly reduced the incidence of MACE across multiple studies. Agarwal et al. reported a hazard ratio (HR) of 0.86 (95% CI: 0.78-0.95; P = 0.0018), indicating a significant reduction in MACE [7]. In the Asian subgroup, Koya et al. found that finerenone reduced the risk of adverse cardiovascular events with an HR of 0.85 (95% CI: 0.59-1.21) [6]. Pitt et al. demonstrated that in diabetic patients with CKD, finerenone therapy improved cardiovascular outcomes with an HR of 0.87 (95% CI: 0.76-0.98; P = 0.03) [8]. Similarly, Filippatos et al. observed a reduced incidence of MACE with an HR of 0.86 (95% CI: 0.75-0.99), with no significant differences based on preexisting CVD status [9]. Bansal et al. noted that finerenone’s benefits were consistent across age and sex categories, with no significant differences in treatment effects, by reporting an HR of 0.86 (95% CI: 0.78-0.95) [10]. Filippatos et al., in their subgroup analysis, reported improved cardiorenal outcomes in patients with and without heart failure, with no significant differences in effect between subgroups [11]. Furthermore, Filippatos et al., in the FIDELITY pooled analysis, found that finerenone reduced the risk of cardiovascular and kidney outcomes consistently across the spectrum of CKD in patients with T2DM, irrespective of atherosclerotic cardiovascular disease (ASCVD) status [12].

Discussion

CKD is a huge public health issue, impacting around 10% of the global population [1]. Its prevalence increases with age and is particularly common among individuals with T2DM and CVD. The Global Burden of Disease study points out that CKD ranks as the 12th leading cause of death in the world and is expected to become the fifth leading cause by 2040 [15]. In the United States alone, nearly 37 million people have CKD, with millions more at risk due to comorbid conditions such as T2DM and CVD [16]. CKD significantly elevates the risk of cardiovascular morbidity and mortality, making it a critical area of focus in public health and clinical practice.

CKD is defined as a persistent abnormality in kidney structure or function lasting over three months. This condition includes one or more of the following criteria: a GFR below 60 mL/min/1.73 m², urine albumin of 30 mg or more per 24 hours, or a urine albumin-to-creatinine ratio (ACR) of 30 mg/g or more, abnormalities in urine sediment, kidney histology, or imaging that indicate kidney damage, renal tubular disorders, or a history of kidney transplantation [17].

The pathophysiology of CKD involves complex interactions among metabolic, hemodynamic, and inflammatory factors. In patients with T2DM, chronic hyperglycemia activates several pathogenic pathways, including the polyol pathway, protein kinase C pathway, and the formation of advanced glycation end-products. These pathways contribute to oxidative stress, endothelial dysfunction, and inflammation, leading to glomerular hyperfiltration, increased glomerular basement membrane thickness, and mesangial expansion. Additionally, hyperglycemia-induced activation of the renin-angiotensin-aldosterone system (RAAS) exacerbates hypertension and promotes further kidney damage. Over time, these changes result in glomerulosclerosis and tubulointerstitial fibrosis, the final common pathways of CKD [18].

According to the KDIGO 2024 Clinical Practice Guideline for the Evaluation and Management of Chronic Kidney Disease, current treatment guidelines for CKD emphasize controlling the causative factors such as diabetes and hypertension, reducing proteinuria, and slowing the progression of kidney disease [19]. Primary pharmacological treatments include angiotensin-converting enzyme inhibitors (ACEi) and angiotensin II receptor blockers (ARBs), which are the cornerstone of CKD management, particularly in patients with proteinuria. These drugs inhibit the RAAS, thereby reducing blood pressure, proteinuria, and progression of kidney disease. However, their use is limited by the risk of hyperkalemia and renal function deterioration. Sodium-glucose cotransporter-2 inhibitors (SGLT2i) have shown promise in reducing CKD progression in patients with T2DM. These drugs lower blood glucose levels and have been demonstrated to reduce cardiovascular events and slow kidney function decline. However, their use can cause adverse effects such as genital infections and euglycemic diabetic ketoacidosis [20]. Steroidal MRAs like spironolactone and eplerenone manage hypertension and reduce proteinuria in CKD patients but are limited by significant side effects, including hyperkalemia [21]. This limitation has driven the development of non-steroidal MRAs like finerenone, which aim to provide similar benefits without adverse effects.

Patients with CKD are at a greater risk of major cardiovascular events, like myocardial infarction, stroke, heart failure, and cardiovascular death. The interplay between CKD and CVD is multifaceted, involving shared risk factors such as hypertension, diabetes, and dyslipidemia, as well as CKD-specific factors like anemia, hyperphosphatemia, and increased oxidative stress. CKD also leads to structural and functional changes in the heart and vasculature, such as left ventricular hypertrophy and arterial stiffness, predisposing patients to adverse cardiovascular outcomes [2]. Therefore, managing cardiovascular risk in CKD patients is crucial.

Finerenone is a novel non-steroidal MRA developed to overcome the limitations of traditional steroidal MRAs. It belongs to selective MRAs. It blocks aldosterone binding to mineralocorticoid receptors, mitigating aldosterone-induced sodium retention, inflammation, and fibrosis, critical drivers of CKD progression and cardiovascular damage [22]. Finerenone’s higher selectivity for the mineralocorticoid receptor translates to fewer off-target effects and a lower risk of hyperkalemia. It modulates gene expression in the kidney and heart, reducing pro-fibrotic and pro-inflammatory signaling pathways. Preclinical studies have shown that finerenone effectively reduces albuminuria, inflammation, and fibrosis, supporting its potential therapeutic benefits in CKD and CVD patients [23].

This systematic review aimed to evaluate the beneficial effects of finerenone on reducing MACE in patients with CKD and T2DM. We synthesized evidence from multiple studies to determine whether finerenone can significantly improve cardiovascular outcomes compared to current treatment standards. The studies included in this review consistently proved finerenone’s efficacy in reducing cardiovascular events and improving renal outcomes in CKD and T2DM patients.

Effect on MACE

The findings from this systematic review show that finerenone significantly reduces the incidence of MACE in patients with CKD and T2DM. The FIDELITY study by Filippatos et al. showed a significant reduction in MACE with a HR of 0.83 (95% CI: 0.74- 0.94) in patients with ASCVD [12]. Moreover, this reduction was consistent across patients without a history of ASCVD as well, with an HR of 0.91 (95% CI: 0.78-1.06). Furthermore, a subgroup analysis within the same study by Filippatos et al. demonstrated a reduction in MACE irrespective of heart failure status [11].

The study by Koya et al. in an Asian population found that finerenone reduced the risk of adverse cardiovascular events with an HR of 0.85, indicating a 15% reduction in risk compared to placebo (95% CI: 0.59-1.21) [6]. Similarly, Agarwal et al. reported an HR of 0.86, indicating a 14% lower risk of MACE (95% CI: 0.75-0.95; P = 0.0018) [7]. Bansal et al. showed consistent cardiovascular benefits of finerenone across different ages and sexes with an HR of 0.86 (95% CI: 0.78-0.95), with a high p-interaction value (≥0.05), indicating no significant variation in treatment effect [10].

Individual Cardiovascular Outcomes

Finerenone also showed significant benefits in reducing specific cardiovascular outcomes. For instance, it was associated with a significant reduction in hospitalization for heart failure. Filippatos et al. showed that finerenone consistently reduced the risk of cardiovascular outcomes, including hospitalization for heart failure [11]. The HR for heart failure hospitalizations was 0.73 (95% CI: 0.50-1.06) in patients with a history of HF and HR 0.90 (95% CI 0.77-1.04) in patients without a history of HF. A p-interaction value of 0.33 proved that the presence of preexisting HF did not change the outcome and that finerenone reduced hospitalization rates in both populations. The study by Filippatos et al. showed that finerenone greatly reduced the risk of hospitalization for heart failure in patients with a history of ASCVD [12]. The HR for heart failure hospitalizations was 0.82 (95% CI: 0.71-0.94) in patients with a history of ASCVD and 0.86 (95% CI: 0.71-1.04) in those without​, once again, the benefits of finerenone were applicable to both cohorts of patients. Agarwal et al. demonstrated a 22% reduction in hospitalizations for heart failure with a 95% CI of (0.66-0.92), a significant contributor to the overall reduction in cardiovascular events [7].

Subgroup Analyses Based on Ethnicity

Koya et al. explored the efficacy of finerenone in an Asian population, concluding that the benefits observed in the broader population also apply to this subgroup. In Asian patients, an HR of 0.85 (95% CI: 0.59-1.21) was observed. For the rest of the world, an HR of 0.86 (95% CI: 0.74-1.00) was observed. The p-interaction value of 0.95 shows that these results support the wide-scale applicability of finerenone treatment across diverse patient populations [6].

Renal Outcomes

The renal protective functions of finerenone deserve mention in our systematic review. Many of our studies also reported the positive effects of finerenone on the kidney [6-8,10-12].

Koya et al. reported a slower rate of estimated GFR (eGFR) decline in the finerenone group compared to placebo in Asian patients, highlighting its reno-protective effects. This result was also applicable to the rest of the world due to the high p-interaction value [6]. Agarwal et al. demonstrated a composite kidney outcome with an HR of 0.77 (95% CI: 0.67-0.88; p=0.0002) [7]. Similar outcomes were seen with Pitt et al. [8]. Bansal et al. showed consistent renal protective effects across age and sex with an HR of 0.77 (95% CI: 0.67-0.88), similar to Agarwal et al. [7,10]. Filippatos et al. showed that finerenone consistently reduced the risk of adverse renal outcomes. The HR for composite kidney outcomes was 0.79 (95% CI: 0.52-1.20) in patients with a history of HF and HR 0.83 (95% CI 0.73-0.94) in patients without a history of HF [11]. Similar results were seen in Filippatos et al., which compared renal outcomes with ASCVD as a basis [12].

Safety and Adverse Events

The safety profile of finerenone was consistent across the included studies, with a similar incidence of adverse events compared to the placebo group. The most common adverse event was hyperkalemia, which was managed effectively without significant differences in discontinuation rates between the treatment and control groups [7-9]. Agarwal et al. showed a 1.7% treatment discontinuation because of hyperkalemia, which was 0.6% in the placebo group [7]. It also reported a 14% incidence of non-fatal adverse events with finerenone and 6.9% in the placebo group. According to Pitt et al., the incidence of hyperkalemia was 10.8% in the finerenone group [8]. However, hyperkalemia, being the reason for discontinuation of treatment, was only 1.2%. Filippatos et al. showed that hyperkalemia-related discontinuation of treatment was at 2.3% in patients with CVD and 2.2% in patients without CVD, thereby showing no considerable difference [9]. The safety profile of finerenone demonstrates a manageable incidence of hyperkalemia, with similar discontinuation rates compared to placebo, highlighting its safety and tolerability in patients with CKD and CVD.

Comparison With Meta-Analyses

Of the nine studies in this review, two were meta-analyses [13,14]. Jyotsna et al. included seven RCTs with a total of 39,995 participants, focusing on cardiovascular and renal outcomes [13]. This meta-analysis concluded that finerenone significantly reduced the risk of cardiovascular and renal-related mortality compared to placebo, with a relative risk of 0.86, with a 95% CI between 0.80 and 0.93, with a p-value of 0.0002. Similarly, the meta-analysis by Abdelazeem et al. analyzed three RCTs with a total of 13,847 patients and found that finerenone was associated with significantly lower rates of cardiovascular events compared to placebo with an RR of 0.88, with a 95% CI between 0.80 and 0.96, with a p-value < 0.01 [14]. Our systematic review supports the findings of these meta-analyses, demonstrating that finerenone effectively reduces MACE.

Limitations

This systematic review has several limitations. First, the heterogeneity among the included studies in terms of study design, patient populations, and outcome measures may introduce variability in the results. Although meta-analytic techniques were used to account for this heterogeneity, some residual variability is inevitable. Second, the relatively short follow-up periods in some studies may limit the ability to capture long-term cardiovascular and renal outcomes. Thirdly, relying on published data may introduce publication bias since studies with negative or inconclusive results are less likely to be published. Finally, while finerenone shows promise in reducing cardiovascular events and improving renal outcomes, its long-term safety and efficacy need further investigation through extended follow-up studies and real-world evidence.

## Conclusions

In conclusion, this systematic review and meta-analysis provide strong evidence supporting the efficacy and safety of finerenone in patients with CKD and T2DM. Finerenone significantly reduces major cardiovascular events, particularly hospitalizations for heart failure, and improves renal outcomes by reducing albuminuria and slowing the decline in eGFR. Despite the limitations, these findings highlight the potential of finerenone as a valuable addition to the therapeutic landscape for managing CKD and T2DM. Future studies should address the unanswered questions, including the long-term benefits and safety of finerenone, and explore its efficacy in diverse patient populations.
